# Introductory Lectures

**Published:** 1848-02

**Authors:** 


### INTRODUCTORY LECTURES.

Lecture Introductory to a course on the Principles and Practice of Surgery in the Jefferson Medical College, delivered November 1st, 1847. By Thomas D. Mütter, M. D. etc. Published by the Class.

Lecture Introductory to the course on the Practice of Medicine in the Jefferson Medical College, delivered November 2d, 1847. By J. K. Mitchell, M. D., etc. Published by the Class.

An Introductory Lecture, delivered to the Class of Institutes of Medicine in Jefferson Medical College, November 4th, 1847. By Robley Dunglison, M. D. Published by the Class.

Memoir of George McClellan, M. D. A Lecture Introductory to the Course of the Theory and Practice of Physic in the Medical Department of Pennsylvania College, for the Session of 1847–48. By William Darrach, M. D. Published by the Class.

Introductory Lecture delivered in the Philadelphia College of Medicine, Session of 1847–48. By James McClintock, M. D., Professor of Surgery and Anatomy. Published by the Class.

Introductory Lecture delivered at the Winter Session of the Philadelphia College of Medicine, November, 1847. By Jesse R. Burden, M. D., Professor of Materia Medica and Therapeutics. Published by the Class.

An Address delivered at the Castleton Medical College, Introductory to the Autumnal Session of 1847. By William Sweetser, M. D., Professor of the Theory and Practice of Medicine. Published by the Class.

